# Imaging appearance on CT post laparoscopic Roux-en-Y gastric bypass using bioabsorbable prosthesis with ﬁbrin glue ﬁxation to prevent a Petersen’s space hernia

**DOI:** 10.1259/bjrcr.20180111

**Published:** 2019-04-09

**Authors:** Mark Page, James Drummond, Mark Magdy, John Vedelago, Vytauras Kuzinkovas

**Affiliations:** 1 Department of Radiology, Tel Aviv Sourasky Medical Center, Israel; 2 Everlight Radiology, SydneyNSW, Australia; 3 Department of Surgery, St George Private Hospital, Sydney, Australia; 4 Department of Radiology, St Mary’s Hospital, London, UK

## Abstract

Imaging post bariatric surgery is becoming more common over the past decade due to increasing incidence of obesity in the population and subsequent treatment.

In recent years, the use of topical haemostatic agents and bioabsorbable prostheses has increased leading to higher likelihood of encountering these agents on post-operative imaging. Imaging in the post-operative period is occasionally performed to assess for complications such as obstruction, leak and abscess formation. Familiarity with these agents is crucial in preventing incorrect diagnosis.

Laparoscopic Roux-en-Y gastric bypass (RYGB) is favoured over the open approach as it is safer and more effective, with a mortality rate of 0.5% and morbidity around 7–14 %. The main cause of late post-RYGB complications is the development of internal hernias such as a Petersen’s hernia. During the procedure, a space between the alimentary loop of the small bowel and the transverse mesocolon is created and is called the Petersen’s defect. Subsequently, a part of the small bowel can herniate through this orifice. As this operation is becoming more common, the incidence of internal herniation has been increasing.

This case report describes a new bariatric surgical technique and the associated post-operative radiological appearances on CT. The surgical technique has been pioneered in Sydney, Australia and involves a laparoscopic RYGB using bioabsorbable prosthesis with ﬁbrin glue ﬁxation to prevent a Petersen’s space hernia.

Imaging post-bariatric surgery is becoming more common over the past decade due to increasing incidence of obesity in the population and subsequent treatment.^1^

In recent years, the use of topical haemostatic agents and bioabsorbable prostheses has increased leading to higher likelihood of encountering these agents on post-operative imaging.^2^ Imaging in the post-operative period is occasionally performed to assess for complications such as obstruction, leak and abscess formation. Familiarity with these agents is crucial in preventing incorrect diagnosis.

Laparoscopic Roux-en-Y gastric bypass (RYGB) is favoured over the open approach as it is safer and more effective, with a mortality rate of 0.5% and morbidity around 7–14%.^3^ The main cause of late post-RYGB complications is the development of internal hernias such as a Petersen’s hernia.^4^ During the procedure, a space between the alimentary loop of the small bowel and the transverse mesocolon is created and is called the Petersen’s defect. Subsequently, a part of the small bowel can herniate through this orifice. As this operation is becoming more common, the incidence of internal herniation has been increasing.^3^

This case report describes a new bariatric surgical technique and the associated post-operative radiological appearances on CT. The surgical technique has been pioneered in Sydney, Australia^5^ and involves a laparoscopic RYGB using bioabsorbable prosthesis with ﬁbrin glue ﬁxation to prevent a Petersen’s space hernia.

## Ethical approval

Written informed consent was obtained from the patient in regards to the publication of this case report and associated images.

### Surgical technique

**Figure 1.**
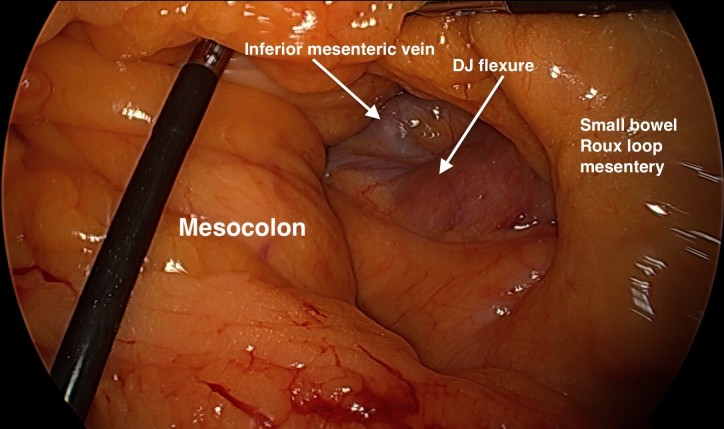
Photograph of Petersen’s defect laparoscopically following Roux-En-Y gastric bypass.

**Figure 2.**
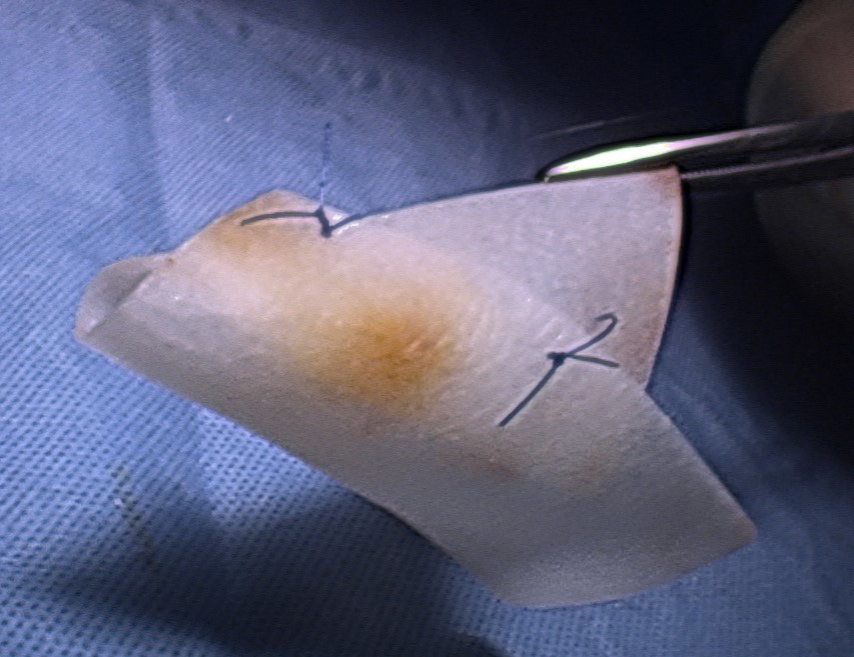
Photograph of the Gore BioA tissue reinforcement mesh

**Figure 3.**
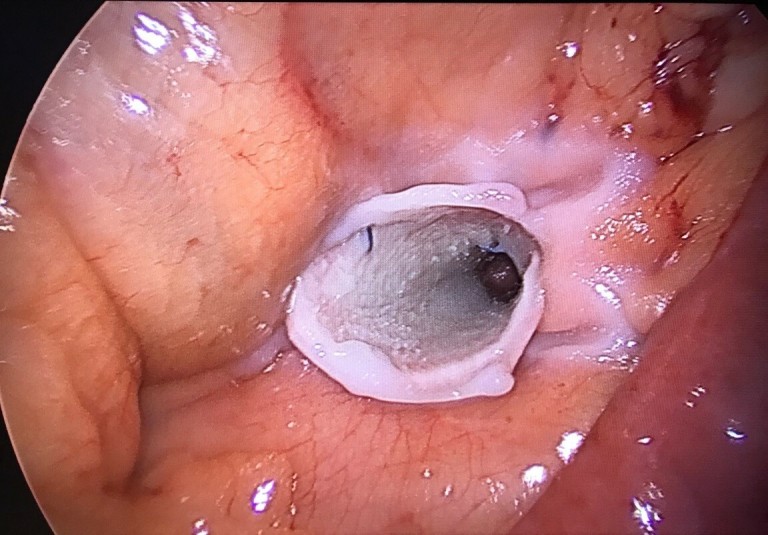
Photograph of Petersen’s defect containing the Gore BioA tissue reinforcement mesh and glue

A standard Laparoscopic RYGB is performed, where the gastric pouch was created with an endoscopic stapler. Following this, the jejunum is transected and an antecolic single layer handsewn gastroenterostomy is performed. A single layer handsewn enteroentrostomy is then performed. Petersen’s space is then inspected as per Figure 1. Following this procedure, a 1 mm Gore BioA Tissue Reinforcement (HH0710) mesh is rolled into a funnel shape and then the roll’s shape is fixed with two interrupted 2/0 Prolene sutures (Figure 2). The rolled mesh is then placed (not sutured) into a Petersen’s defect as a plug. Tissel Glue is sprayed around the mesh edges to help it stick to the surrounding bowel mesenteries, so establishing a “matrix” for a better tissue collagen ingrowth/integration into the mesh (Figure 3).

### Appearance on CT

**Figure 4.**
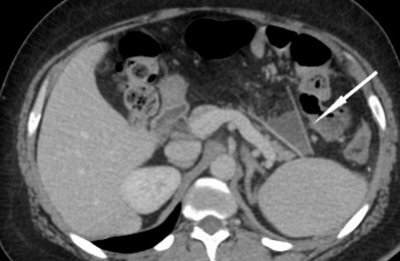
Axial CT post-i.v. contrast 1 week post laparascopic Roux-en-Y gastric bypass using bioabsorbable prosthesis with ﬁbrin glue ﬁxation to prevent a Petersen’s space hernia. Funnel shaped structure (arrow) in Peterson’s defect corresponds to the Gore BioA tissue reinforcement mesh

**Figure 5.**
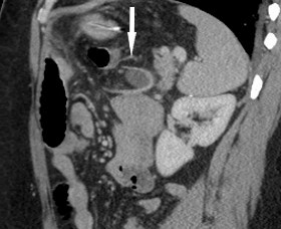
Sagittal CT post i.v. contrast 1 week post laparascopic Roux-en-Y gastric bypass using bioabsorbable prosthesis with ﬁbrin glue ﬁxation to prevent a Petersen’s space hernia. Funnel shaped structure (arrow) in Peterson’s defect corresponds to the Gore BioA tissue reinforcement mesh

**Figure 6.**
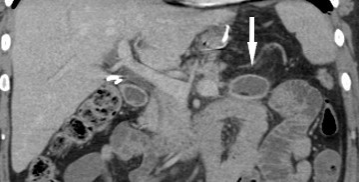
Coronal CT post-i.v. contrast 1 week post laparascopic Roux-en-Y gastric bypass using bioabsorbable prosthesis with ﬁbrin glue ﬁxation to prevent a Petersen’s space hernia. Funnel shaped structure (arrow) in Peterson’s defect corresponds to the Gore BioA tissue reinforcement mesh

CT with i.v. contrast performed 1 week post-procedure due to the nonspecific abdominal pain (turning out to be related to constipation) demonstrated status post-RYGB with a funnel shaped structure within the Petersen’s defect opening anteromedially to the right with the apex pointing posterolaterally towards the spleen. The structure was cone/funnel shaped, it had a linear dense sharp margins and contained dependent fluid and fat centrally as seen in Figures 4–6. There is no gas entrapment as seen with other bioabsorbable prostheses. There is no rim enhancement within the fluid or in the opening anteriorly. No evidence of bowel herniating into the structure. No metallic density. The appearances are not typical for an abscess as there is no rim enhancement or internal gas. The appearances are not typical for a surgical foreign body as there is no metallic artefact or entrapped gas within a soft tissue density to suggest presence of gauze. The appearances correspond to the funnel shaped prosthesis seen in Figure 1 with dependent fluid filling the centre.

## Discussion

This technique has been performed for 16 months. To date 71 Laparoscopic RYGB bypasses, using bioabsorbable prosthesis with ﬁbrin glue ﬁxation to prevent a Petersen’s space hernia have been performed. No significant postoperative complications or hernias associated with this prosthesis have been reported in this cohort.

A few patients in the cohort required laparoscopies for unrelated reasons at 3 months after RYGB, one patient had a plastic gastric pouch ring intolerance symptoms and required its removal, another patient had laparoscopy for pelvic adhesions. During these laparoscopies Petersen’s space was inspected, where the previous bioabsorbable prosthesis has been placed. The prosthesis was completely gone and the Petersen’s space was fully obliterated with natural fibrous adhesions, essentially removing any chance of the internal hernia protruding through it.

Seeing the very promising evidence of the mesh being replaced with the solid natural body adhesions, which completely obliterate and solidify the Petersen’s space. In our opinion, it is physically impossible for the mesenteric defects to widen, even if the patient loses weight.

Recent years have witnessed the use of bioabsorbable and biologic agents for topical haemostasis in the operative field. The Gore BioA, material will be absorbed within 6 months, leaving no permanent material behind in the body. The majority of the other agents are expected to resolve within 3–6 months, however, in some instances they can last for years due to a chronic inflammatory response. The appearances of these agents is variable on imaging and is based on their intrinsic characteristics. These agents can have fat, soft tissue and fluid density on CT with occasional entrapped gas within the matrix (2).

The Gore BioA is composed of 67% polyglycolic acid and 33% trimethylene carbonate. It provides a scaffold for inflammatory response cells to infiltrate including fibroblasts. This eventually leads to dense collagen deposition which replaces the resorbing scaffold. This explains the appearance in the early post-operative period with linear, homogenous well-defined margins of the mesh that does not contain internal gas, fat or fluid. Whereas the small amount of post-operative fluid and fat is in the centre of the funnel shape surrounded by the mesh.

This is the first radiological description of post-operative findings, related to this particular surgical technique. It is important for the radiologist to become familiar with this appearance to prevent misdiagnosis such as abscess formation or a gossypiboma. Knowledge of the procedure performed and communication with the surgeon are imperative to ensure the correct diagnosis.

### Learning points

- The use of topical haemostatic agents and bioabsorbable prostheses has increased.
- Familiarity with their appearance is crucial to prevent misdiagnosis such as abscess formation or a gossypiboma
- Laparoscopic RYGB using bioabsorbable prosthesis with ﬁbrin glue ﬁxation to prevent a Petersen’s space hernia is a technique gaining popularity.
